# Severe adverse effect of antireflux band mucosectomy

**DOI:** 10.1055/a-2615-6258

**Published:** 2025-06-26

**Authors:** Hsin-Yu Hung, Keng-Wei Liang, Edy Kornelius, Hau-Jyun Su, Ming-Chang Tsai, Chi-Chih Wang

**Affiliations:** 1 63276 Division of Gastroenterology and Hepatology, Department of Internal Medicine, Chung Shan Medical University Hospital, South District, Taichung, Taiwan; 2 34899 School of Medicine, Chung Shan Medical University, Taichung, Taiwan; 3 63276 Department of Medical Imaging, Chung Shan Medical University Hospital, Taichung, Taiwan; 4 34899 Institute of Medicine, Chung Shan Medical University, Taichung, Taiwan

A 43-year-old man without underlying disease presented with refractory globus sensation and heartburn for 1 year. Esophagogastroduodenoscopy (EGD) revealed esophagus gastroesophageal reflux disease (GERD), Los Angeles classification grade C with hiatal hernia, Hill grade II. On 96-hour ambulatory esophageal pH monitoring and high-resolution manometry, lower pressure at the lower esophageal sphincter was confirmed, with normal esophageal peristalsis. Therefore, he received antireflux band mucosectomy (ARBM) ( Fig. 1 , Video 1 ) with a multiband ligator.

**Fig. 1:**
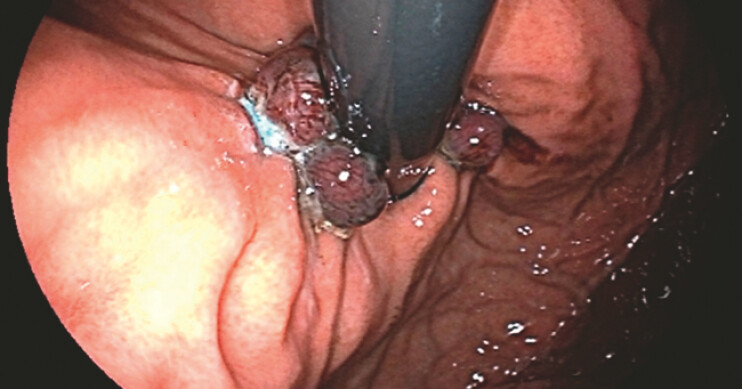
Five ligations were applied at the posterior wall, lesser curvature, and anterior wall of the cardia during antireflux band mucosectomy.

Antireflux band mucosectomy for refractory esophagus gastroesophageal reflux disease.Video 1

However, hematemesis and hypovolemic shock occurred 14 hours post-ARBM. Emergency EGD identified one active spurting site between the greater curvature and the anterior wall of the cardia, and endoscopic hemostasis was performed ( Fig. 2 ). Hypovolemic shock persisted (59/36 mmHg) despite the use of inotropes and a Sengstaken–Blakemore tube. Dynamic abdominal computed tomography revealed a left gastric artery pseudoaneurysm at the esophagogastric junction ( Fig. 3 ), which was subsequently embolized via emergency transcatheter arterial embolization ( Fig. 4 ), stabilizing the hypovolemic shock. The patient received a total of 4000 mL packed red blood cells, 3000 mL fresh frozen plasma, and 800 mL platelet pheresis transfusion during resuscitation. The patient was discharged 12 days after ARBM without any permanent sequelae.

**Fig. 2:**
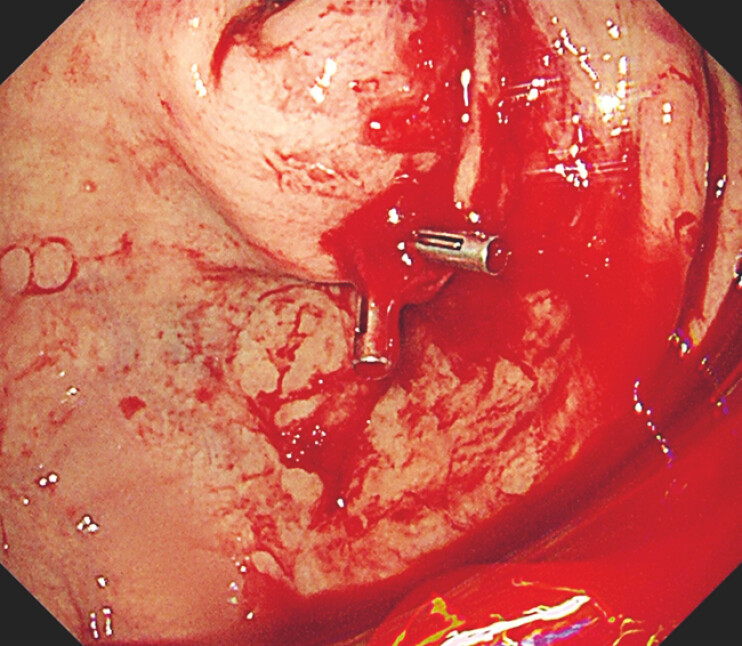
Endoscopic hemostasis was performed at the bleeding site between the greater curvature and the anterior wall of the cardia.

**Fig. 3:**
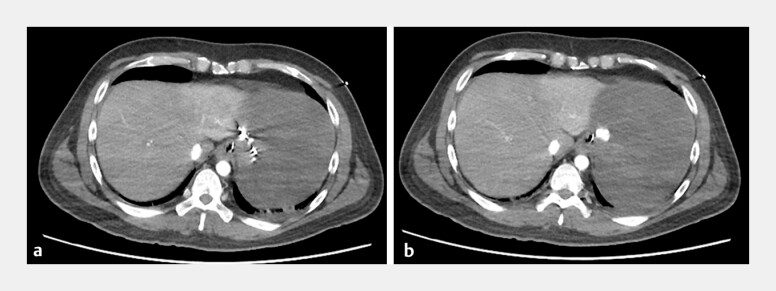
Dynamic abdominal computed tomography revealed a left gastric artery pseudoaneurysm at the esophagogastric junction.

**Fig. 4:**
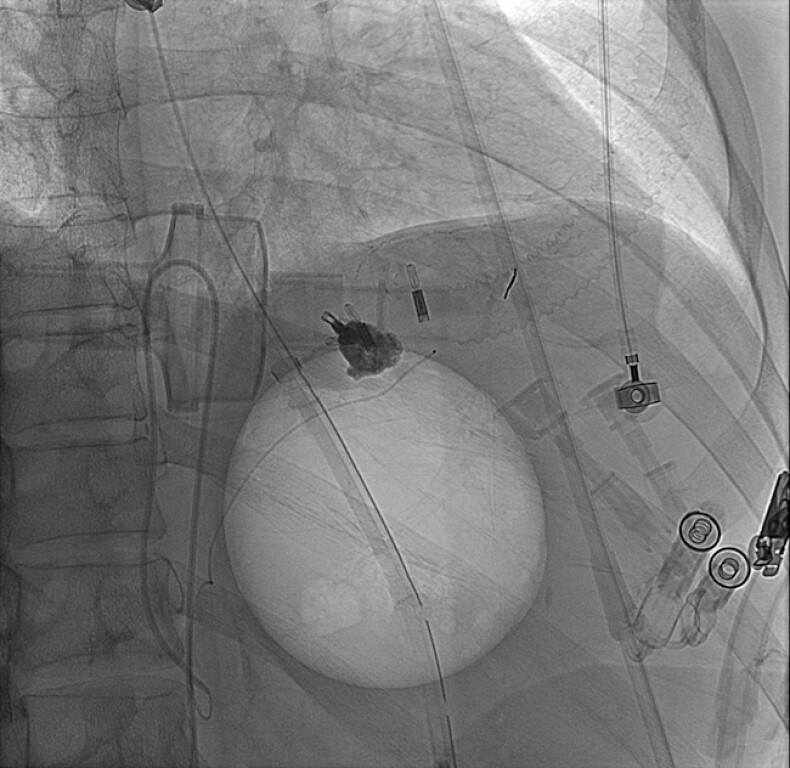
Transcatheter arterial embolization of the left gastric artery pseudoaneurysm was performed.

ARBM is an effective alternative method of mucosectomy for refractory GERD using endoscopic mucosal band ligation devices 1 2 . A previous study, which included patients between June 2017 and January 2019, revealed only one case (4.8%) of nonsevere bleeding, without blood unit transfusion, endoscopic treatment, or hospitalization, as a postoperative adverse event 3 . Further meta-analysis study reported no immediate bleeding after ARBM 4 . Hence, it was suggested that ARBM can be performed in the ambulatory setting. However, our case demonstrates a life-threatening bleeding complication, such as left gastric artery pseudoaneurysm rupture. Our case emphasizes that the risk of severe bleeding with ARBM should not be ignored, and further large-scale investigations are needed to determine the safety profile.

Endoscopy_UCTN_Code_CPL_1AH_2AH

## Notes

Endoscopy E-Videos https://eref.thieme.de/e-videos
*E-Videos* is an open access online section of the journal *Endoscopy* , reporting on interesting cases and new techniques in gastroenterological endoscopy. All papers include a high-quality video and are published with a Creative Commons CC-BY license. Endoscopy E-Videos qualify for HINARI discounts and waivers and eligibility is automatically checked during the submission process. We grant 100% waivers to articles whose corresponding authors are based in Group A countries and 50% waivers to those who are based in Group B countries as classified by Research4Life (see: https://www.research4life.org/access/eligibility/ ). This section has its own submission website at https://mc.manuscriptcentral.com/e-videos .
